# Design of a loading system for cyclic test on sutured organs

**DOI:** 10.1016/j.mex.2020.100988

**Published:** 2020-07-08

**Authors:** Giulia Pascoletti, Maria Chiara Pressanto, Giovanni Putame, Mara Terzini, Giordano Franceschini, Elisabetta M. Zanetti

**Affiliations:** a Department of Engineering, University of Perugia, Italy; b Dipartimento di Medicina Veterinaria - Sezione Chirurgia e Radiodiagnostica, University of Perugia, Italy; c Polito^BIO^Med Lab, DIMEAS, Politecnico di Torino, Italy

**Keywords:** Suture test, Suture distraction, Cyclic loads, Anchorage points migration

## Abstract

The design of loading systems to test biologic samples is often challenging, due to shape variability and non-conventional loading set-ups. In addition to this, large economic investments would not be justified since the loading set up is usually designed for one single or for a limited range of applications.

The object of this work is the development of a loading set-up finalised to on-site testing of sutures whose main function is applying a localised tensile load. The main challenges of this design process can be so summarized:

• Applying cyclic tensile loads on the suture wire, mimicking the physiologic condition where both suture anchorage points have a certain compliance;
• Designing a loading system as versatile as possible, in order to be able to accommodate organs with different geometries and sizes;
• Keeping low both the complexity and costs of realization.

All these considerations and the design calculi are here reported in detail, discussing the novelty of the system, and its main advantages.

## Graphical abstract

**Figure:**
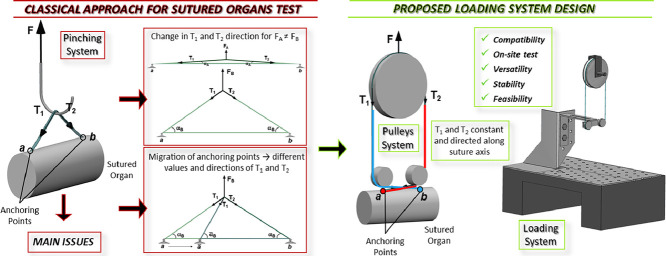

Specifications Table

**Table:** 

Subject Area	Engineering
More specific subject area	*Biomechanics*
Method name	*On-site testing of prosthetic sutures*
Name and reference of original method	
Resource availability	*Technical drawings complete with quotes have been provided as supplementary material for each component designed in this work.*
	*In addition, a video showing the application of the loading system to a laryngoplasty has been provided.*

## Method details

**Fig. 1:**
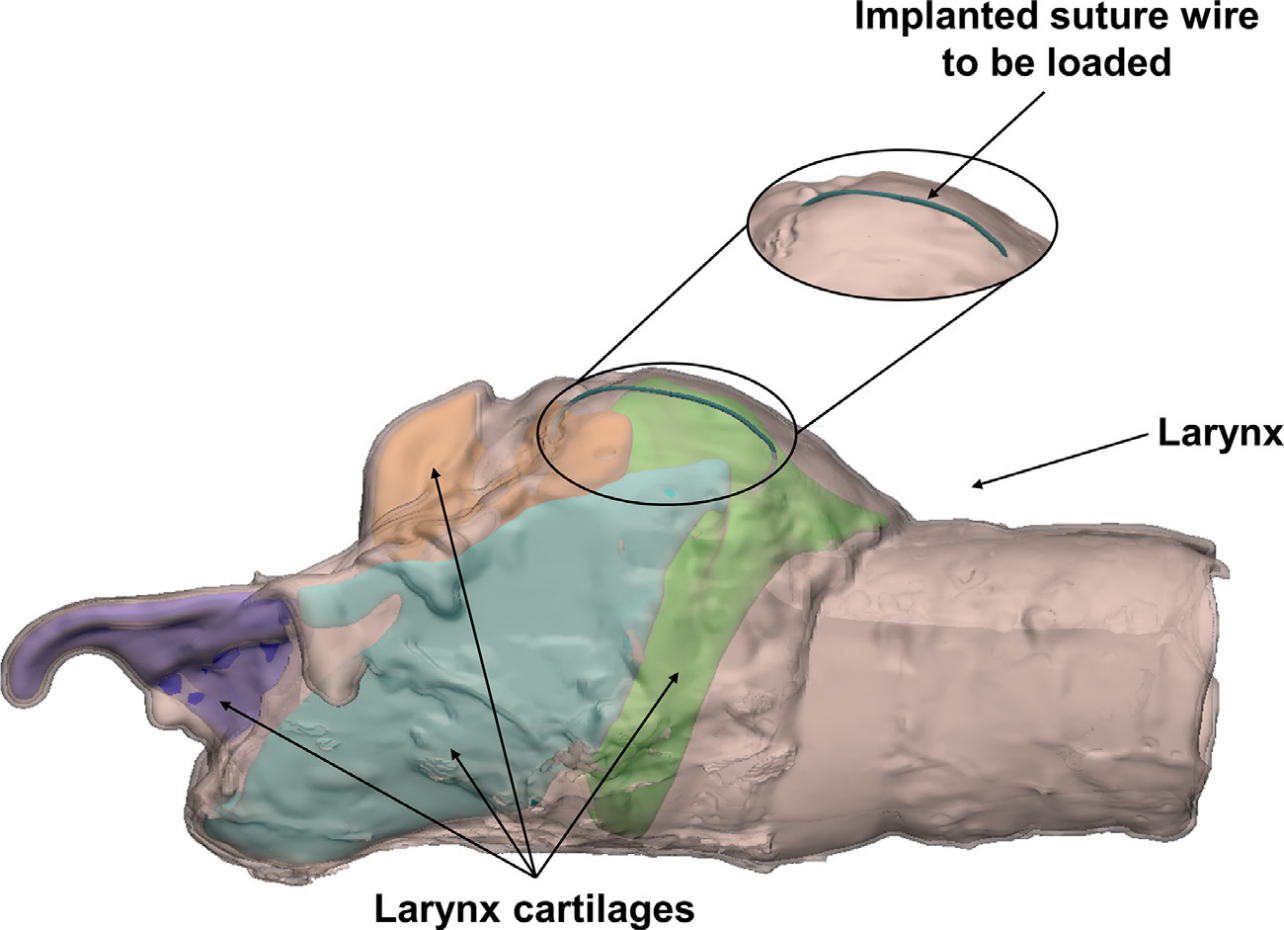
Equine laryngoplasty.

In this methodological work the design of a non-standard loading system is described. The authors have designed a loading system which was intended to perform cyclic tests on sutured organs (Fig. 1).

First of all, its main requirements have been identified, as described in the following.

• *Interfaceability/Compatibility with the testing machine*: The loading system must be interfaceable with a standard testing machine with reference to both its base and its actuator. In this case the testing machine was an Instron Electropulse E3000.
• *Loading the suture along its axis*: The suture had to be loaded along the direction joining its two anchorage points on the organ.
• *Testing the suture on-site*: This requirement came from the necessity of taking into account the compliance of suture anchorage points at both ends.
• *Versatility*: The sutured organ does not have a standard shape (it could be a larynx, a uterus, etc.) and it might show significant anatomic variability among subjects.
• *Stability and stiffness*: The design had to provide an excellent stability and stiffness in order to guarantee a good repeatability of results and to minimize the bias produced by the adopted loading system.
• *Feasibility*: The components should be immediately available or they should be simple to be manufactured.

The first step of the design required the identification of the best way to properly load the suture wire once it has been implanted into the organ.

**Fig. 2:**
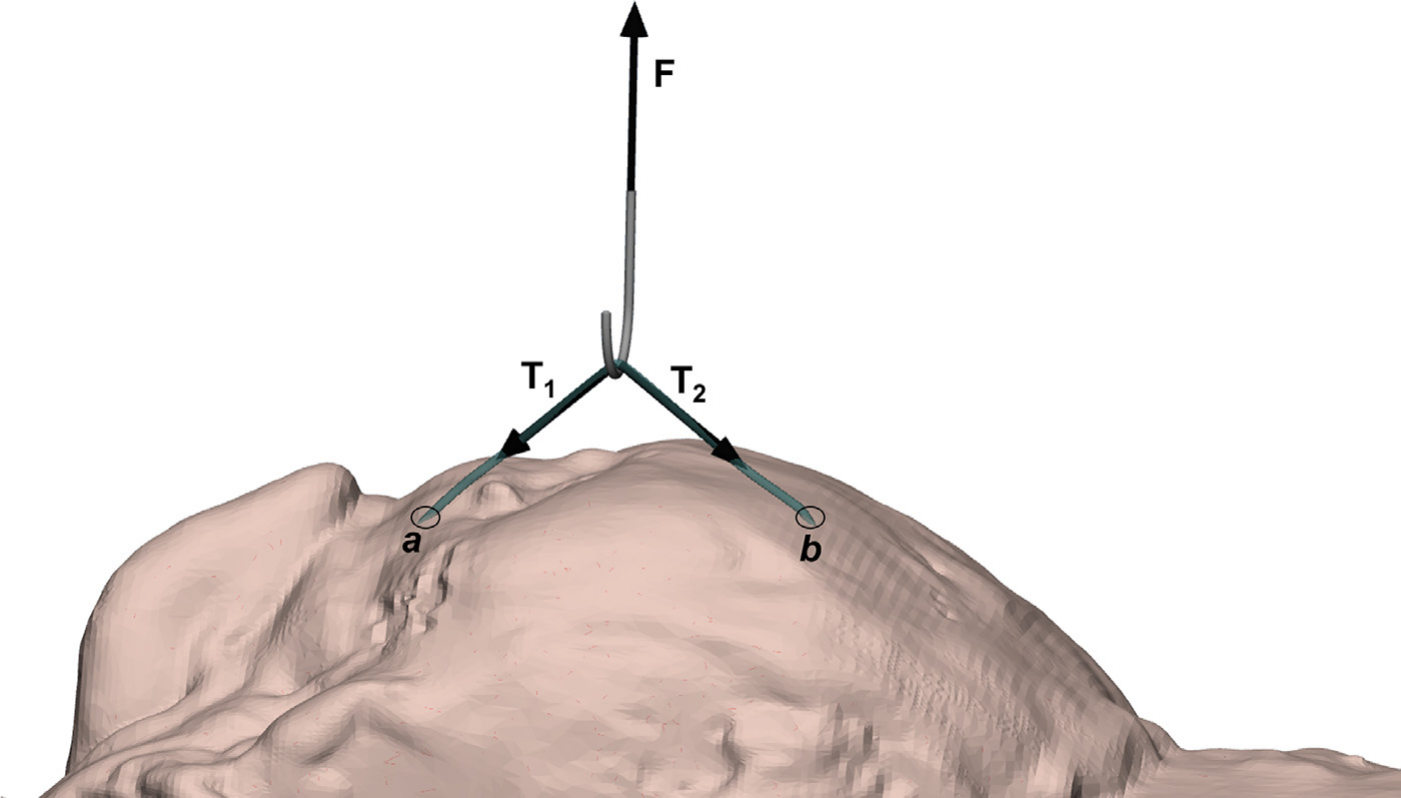
Loading the wire through ‘pinching’.

Loading configurations used in previous works to stress sutures on-site [2], [3], [4], [5] consist in directly pinching the implanted suture wire with the desired cyclic load, as shown in Fig. 2.

This approach exhibits some important issues: →→

**Fig. 3:**
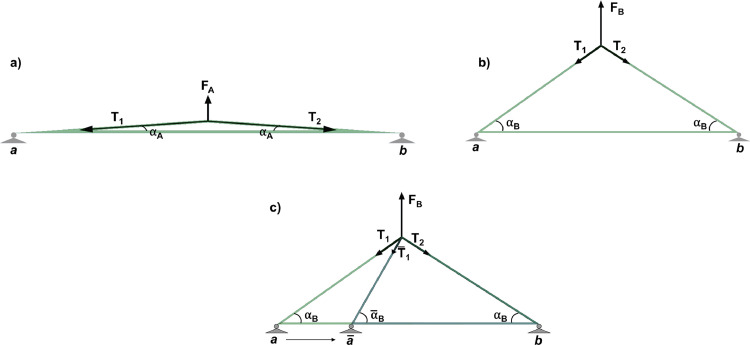
(a), (b) Static equilibrium of forces for two different load levels (F_A_ < F_B_); (c) Static equilibrium of forces in the case of migration of one anchorage point of the suture (point *a*).

1 The direction of forces **T**_**1**_ and **T**_**2**_ applied to the two branches of the suture changes during the test because of the variation of α angle (Fig. 3(a) and (b)). This implies that also the ratio between→→the applied force (F_A_ or F_B_) and **T**_**1**_ and **T**_**2**_ changes during the test, as resulting from equilibrium equations:
  (1) $$\left\{ \begin{array}{c} T_{2}\cos\left(\alpha \right)+T_{1}\cos\left(\alpha \right)=0\;\to \;T_{1}=T_{2}\\ F-T_{1}\sin\left(\alpha \right)-T_{2}\sin\left(\alpha \right)=0\;\to \;T_{1}=T_{2}=\frac{F}{2\text{sin}\left(\alpha \right)} \end{array}\right.$$

The loading machine controls the applied force, while α angle is variable, so the thread tension cannot be controlled.

2 Another problem related to this configuration is the possibility of a different migration of the anchoring points (*a* and *b* in Figs. 2 and 3) of the suture: soft tissues tearing can take place at these points due to the applied load, resulting in suture migration. In the absence of the lower pulleys, this phenomenon would lead to different values and directions of *T*_1_ and *T*_2_ forces (Fig. 3(c)).

**Fig. 4:**
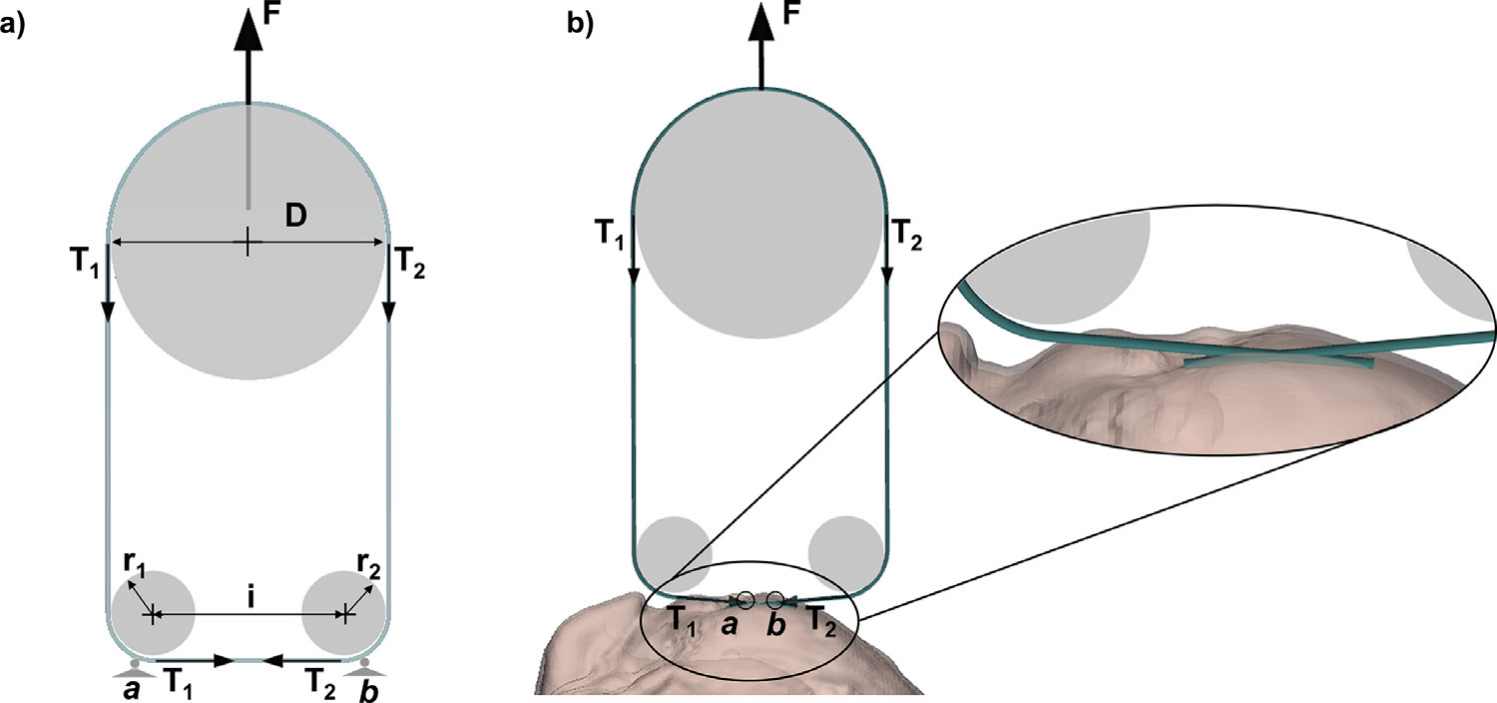
(a) Static equilibrium of forces for the loading set up here introduced; (b) the path of the wire on a sutured organ.

The loading system depicted in Fig. 4 would overcome both limitations. In facts, considering a massless wire, static loads and a negligible friction, the following equations hold:

(2) $$\left\{ \begin{array}{c} T_{1}-T_{2}=0\;\to \;T_{1}=T_{2}\\ F-T_{1}-T_{2}=0\;\to \;T_{1}=T_{2}=\frac{F}{2} \end{array}\right.$$

This means that this loading system allows calculating in every instant the exact value of the tension which acts on the suture once the force applied by the testing machine is known and controlled: when the loading machine applies a force *F*, the wire tension is equal to half this force (*F*/2). In addition, *T*_1_ and *T*_2_ forces are always horizontal, along the suture wire, independently from anchorage point migration. The above-mentioned conditions apply for a specific design where the diameter of the upper pulley is equal to the sum of the radii of the smaller pulleys (*r*_1_ and *r*_2_) and the distance between their centres (*i*):

(3) $$D=r_{1}+r_{2}+i$$

Having established the working principle, that is the ‘conceptual design’, the next step was the detailed design of each component.

**Fig. 5:**
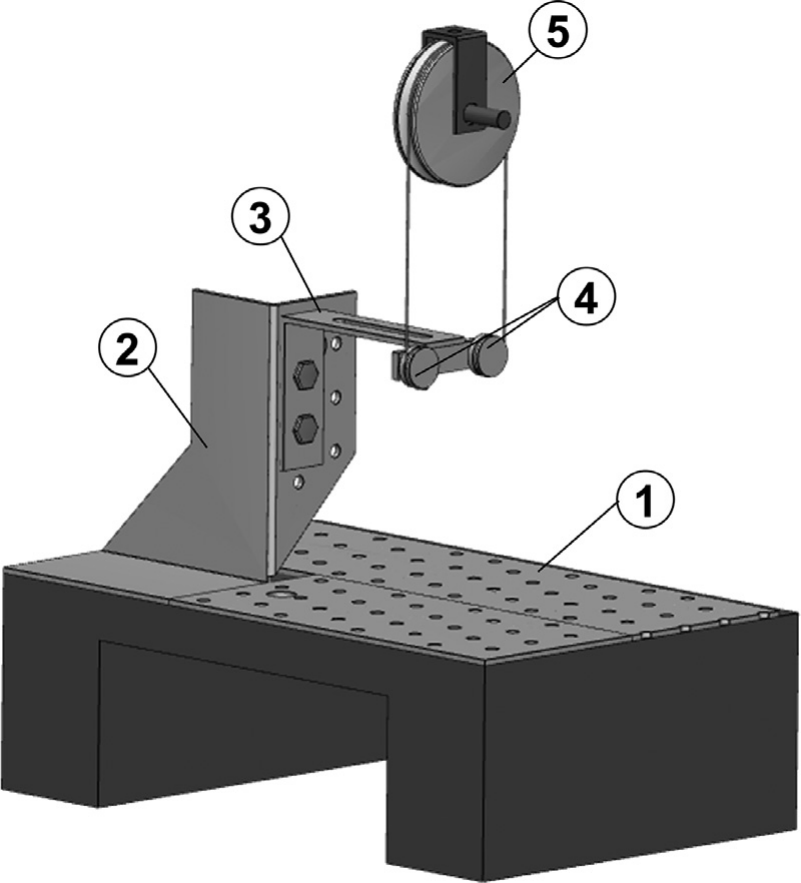
The designed loading system and its components: the support base (1); the support plate (2); the support bar (3); the lower pulleys (4); the upper pulley (5).

The whole loading system is shown in Fig. 5; it is made of the following components:

(1) The Support Base with perforated plates: holes have been made in order to allow constraining the organs through one or more bolts. The support base's weight is over 20 kg in order to provide a good stability: for most applications, it can be simply overlaid on the loading machine base without the need of additional constrains
(2) The Support Plate: this plate has been soldered on the support base and it allows rising the height of the lower pulleys with respect to the support base
(3) The Support Bar with slot: this bar allows regulating the horizontal position of the lower pulleys
(4) The Lower Pulleys: the lower pulleys function is to load the thread along its axis; in order to achieve this aim, their height must be regulated, as visible in Fig. 5
(5) The Upper Pulley: the upper pulley is constrained to the actuator of the loading machine.

All these components will be analysed in detail in the following.

### The support bar and the support plate

Lower pulleys are bolted to the support bar (n.3 in Fig. 2); this component along with the support plate (n.2) makes the whole system adaptable to different organs’ anatomies.

More in detail, the support bar bears a longitudinal slot where the lower pulleys are fixed; this slot allows a fine and continuous tuning of the horizontal position of the pulleys. In addition, the bar bearing the lower pulleys can rotate around a vertical axis. These two regulations guarantee that the line connecting pulleys’ centres and the line passing through the two anchoring points belong to the same vertical plane.

The support bar is connected to the loading system via the support plate; this one is fixed to the support base with three screws, while the support bar is connected to this plate by two bolts. The plate has seven holes which allow adjusting the vertical position of the support bar in relation to the size of the sutured organ.

#### Verification of the maximum displacement of the support bar

The support bar must guarantee a sufficient stiffness when its end is loaded with the peak load, which has been set equal to 100 N. It was calculated admitting 1.5 mm displacement (*δ*) at the point of load application.

According to the well-known equation for cantilever beam load:

(4) $$\delta =\frac{4Fd^{3}}{Ebh^{3}}$$

**Fig. 6:**
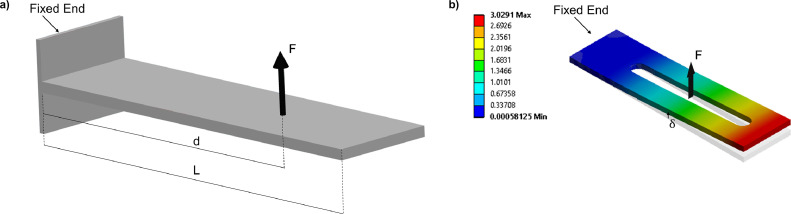
(a) Cantilever load on the support bar; (b) the respective finite element model.

Where *d* is the distance of the load from the beam constrain, *E* is the Young modulus of the material, *b* and *h* are the beam wideness and thickness, respectively (Fig. 6(a)). According to this calculus, given a 20 × 2 mm^2^ bar, *d* must be kept below 50 mm.

Considering that the support bar section bears a slot, a more refined calculus was performed through finite element simulation, which confirmed the analytical finding (Fig. 6(b)).

### The support base

The support base has been designed with the aim of providing stability by its own weight, without the need of nuts and bolts in order to be secured to the lower base of the loading machine. Balance equations for both forces and moments have been considered in order to establish its geometry.

A C-shape has been chosen with the purpose of allowing fastening the organ to the base by means of bolts.

In the following figure the main dimensions of the support base are quoted.

Dimensions *L_b_* and *d_b_* have been defined considering the need of providing a sufficiently wide surface to support the organ and they have been set equal to 250 and 160 mm, respectively.

#### Analysis of stability of the support base

**Fig. 7:**
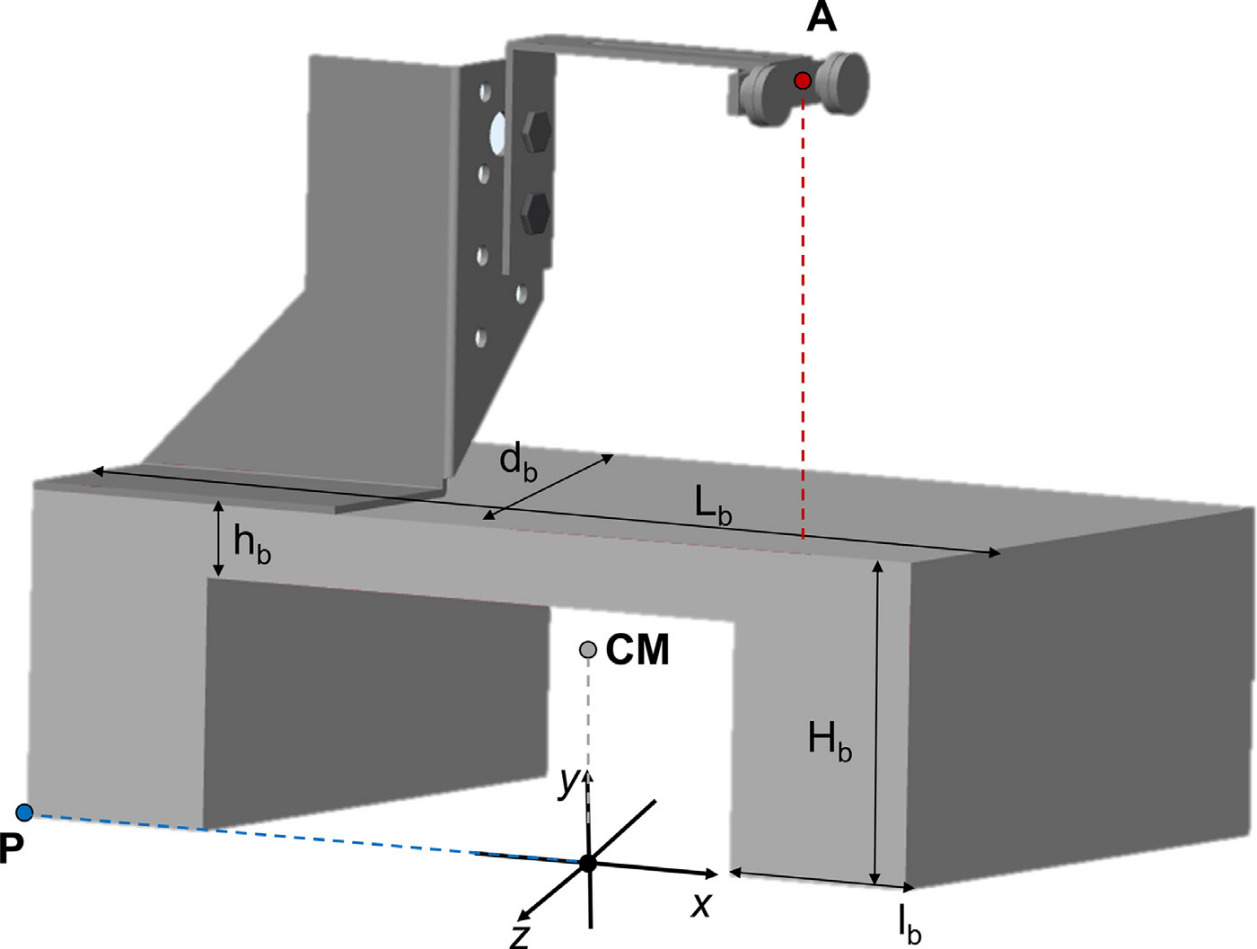
The quoted support base.

In Fig. 7 the following points have been identified:

• CM: it is the centre of mass of the support base; it has coordinates *x_CM_, y_CM_, z_CM_*
• A: it is the point of application of the external force via the pulleys system; it has coordinates *x_A_, y_A_, z_A_*
• P: it is one of the vertices of the base geometry and it is the reference point for the calculation of overturning moments; its coordinates are *x_P_, y_P_, z_P_*

With reference to vertical forces balance, the following equation holds:

(5) $$F_{A}-F_{w}=0\;\to F_{A}=F_{w}$$

where:

- *F_w_* is the force associated to the weight of the support base;
- *F_A_* is the external force applied by the loading machine via the pulleys system; its maximum value being equal to 100 N.

With reference to overturning moments (around the *x* and the *z* axis), the following equations hold, respectively:

(6) $$-F_{w}\left(z_{P}-z_{CM}\right)+F_{A}\left(z_{P}-z_{A}\right)=0$$

(7) $$-F_{w}\left(x_{P}-x_{CM}\right)+F_{A}\left(x_{P}-x_{A}\right)=0$$

In the most unfavourable condition, *A, P*, and *CM* points coordinates are [mm]

$$\left\{ \begin{array}{c} x_{CM}=0\;\\ y_{CM}=47.8\;\\ z_{CM}=0\; \end{array}\right.\;\left\{ \begin{array}{c} x_{A}=42.9\;\\ y_{A}=194.8\;\\ z_{A}=-2.05\; \end{array}\right.\;\left\{ \begin{array}{c} x_{P}=-125\\ y_{P}=0\;\\ z_{P}=80\; \end{array}\right.$$

The weight force of the support base can be so calculated, respectively:

(8) $$-F_{w}\cdot 80+100\cdot 82.05<0\;\;\;F{\;}_{w}>\frac{8205}{80}=102.56N$$

(9) $$-F_{w}\cdot \left(-125\right)+100\cdot \left(-167.9\right)<0\;\;\;\;\;\;F{\;}_{w}>\frac{16790}{125}=134.32N$$

The most restrictive condition is the second one and the minimum mass of the support base can be easily calculated as:

(10) $$M_{b}=\frac{F_{w}}{g}=13.7\;\text{kg}$$

According to above equations, the following dimensions have been chosen for the support base geometry: *L_b_* = 250 mm; *d_b_* = 160 mm; *H_b_* = 80 mm; *h_b_* = 62 mm; *l_b_* = 50 mm. Two perforated plates have been soldered to the support base and their holes have guided base perforation which allowed constraining the organ with bolts.

### Organ constrains

**Fig. 8:**
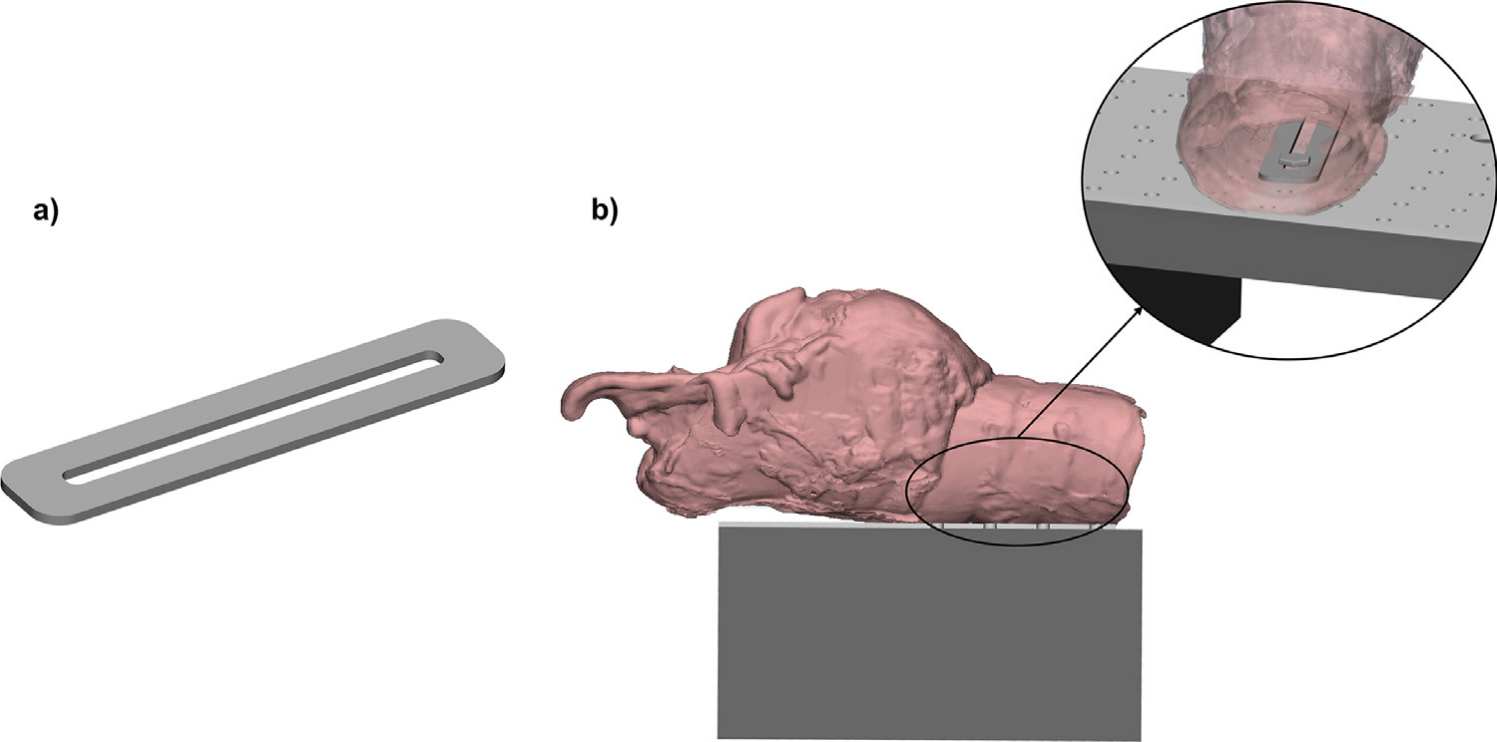
(a) The organ plate; (b) the organ mounted on the support base through its plate.

The suture is connected to testing machine via the pulleys system: the two suture ends are crossed, passed through the lower pulleys and joined above the upper pulley (Fig. 4(b)). The organ is constrained to the loading machine base with two bolts and the plate depicted in Fig. 8; images here reported refer to an equine prosthetic suture called laryngoplasty.

The plate has been put inside the larynx in correspondence of the tracheal rings; two holes are created in the larynx and two screws are passed through the plate and the cartilages. Once the larynx has been correctly positioned on the support base, the bolts are placed: they pass through the plate, the larynx, and the support base.

The presence of multiple holes on the base allows properly orienting the larynx, while the use of the organ plate provides a sufficiently wide support in order to load the specimen without local failures at constrains.

### Alignment

The loading system can work properly only if the upper pulley lies in the same plane as the two lower pulleys and it is centred above them (Fig. 5). This condition can be reached, according to the following procedure:

- The organ is clamped to the support base, as detailed in the previous paragraph;
- The position of the lower pulleys, with respect to the organ, is regulated acting on the height of the support bar, and regulating their horizontal position along the support bar slot;
- The upper pulley is clamped to the moving crosshead of the loading machine;
- The machine crosshead is lowered and the support base is moved so that the upper pulley is aligned with the two lower pulleys. The perfect alignment is checked lowering the machine crosshead and checking that the upper pulley comes into touch with both two lower pulleys (after this check the crosshead is raised to give the desired pre-load).

The cost of all components has reached about 660 Euros.

## Virtual testing

### CAD model

After all the components have been designed, their assembly and their interaction with the larynx have been evaluated numerically. The geometry of single components has been replicated with 3D CAD and the whole system has been assembled.

The three-dimensional model of the organ can be obtained from CT data.

Fig. 5 shows the expected configuration of the loading system; all components have resulted to be properly designed and the organ can be freely oriented on the base plane.

Technical drawings complete with dimensions are provided for each designed component, as supplementary material.

### Multibody model

**Fig. 9:**
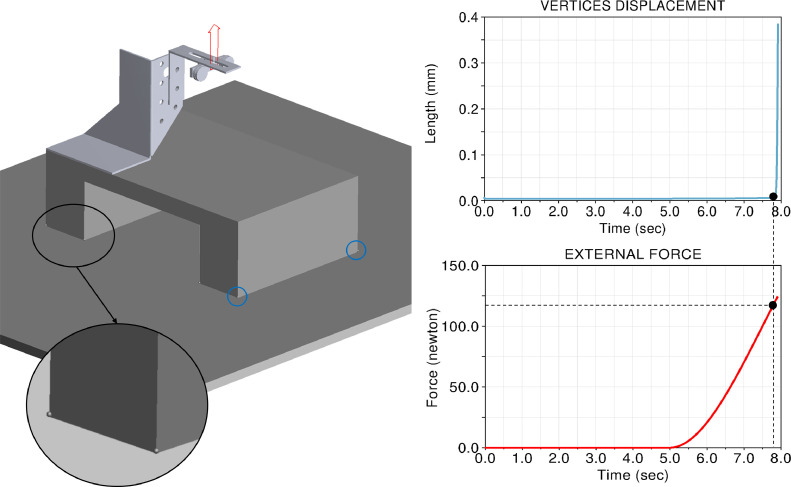
Multibody analysis of the stability of the full system.

A virtual loading test has been performed, considering all elements as solid bodies (Fig. 9).

All elements are modelled as solid bodies, made of steel (density equal to 7800 kg/m^3^); they have been fully constrained, with the only exception of the support base, whose contact with the base of the loading machine has been simulated making use of eight spheres, put at the eight vertices. The simulation has been performed applying the gravitational force and a rising external force from 0 N to 200 N (represented by an arrow in Fig. 9); the force was applied at *d* = 50 mm (Fig. 6a), following a cubic law; the base stability has been checked through eight sensors which control the vertical displacement of the eight spheres.

This simulation has proved that the system is stable, up to 120 N external load. If this load has to be overcome (for example to perform ultimate load tests), the system needs to be clamped to the base of the machine.

## Experimental tests

This loading system has been employed to perform experimental tests on an equine laryngoplasty. In relation to this application, cyclic loads have been applied with displacement control (1 mm/s), between two loads limits (30 N and 50 N), corresponding to physiologic swallowing loads. In the supplementary material, a video is provided where the application of the system to a laryngoplasty procedure is shown. Results obtained on 8 samples are reported extensively in other works by the same authors [1, 6]: the system allowed obtaining consistent results (standard error below 12%) in spite of the natural variability among samples. In addition, in the same article, also ultimate load tests have been carried on with loads reaching 244 N.

## Discussion and limitations

This set up has been designed to test sutured organs behaviour during follow-up, focusing on a relevant complication that is suture loosening [7], resulting from repeated loading cycles. As such, it is not suitable to simulate suture failure when applied loads are perpendicular to the tissue as it may happen during sewing. When the 100 N load limit is overcome, deformations can be relevant; for example, point A in Fig. 7 would be displaced upwardly more than 1.5 mm; consequently, a slightly non-linear behaviour is expected.

## Declaration of competing interest

The authors declare that they have no known competing financial interests or personal relationships that could have appeared to influence the work reported in this paper.

## Appendix Supplementary materials

Supplementary material *and/or* Additional information: Technical drawings complete with quotes have been provided as supplementary material for each component designed in this work, in order allow the replication of the loading system. In addition, a video showing the application of the loading system to a laryngoplasty procedure is provided. Image, video 1

Image, application 2
